# Ligand-Specific Factors Influencing GLP-1 Receptor Post-Endocytic Trafficking and Degradation in Pancreatic Beta Cells

**DOI:** 10.3390/ijms21218404

**Published:** 2020-11-09

**Authors:** Zijian Fang, Shiqian Chen, Yusman Manchanda, Stavroula Bitsi, Philip Pickford, Alessia David, Maria M. Shchepinova, Ivan R. Corrêa Jr, David J. Hodson, Johannes Broichhagen, Edward W. Tate, Frank Reimann, Victoria Salem, Guy A. Rutter, Tricia Tan, Stephen R. Bloom, Alejandra Tomas, Ben Jones

**Affiliations:** 1 Section of Endocrinology and Investigative Medicine, Imperial College London, London W12 0NN, UK; zf250@cam.ac.uk (Z.F.); s.chen18@imperial.ac.uk (S.C.); p.pickford17@imperial.ac.uk (P.P.); v.salem@imperial.ac.uk (V.S.); t.tan@imperial.ac.uk (T.T.); s.bloom@imperial.ac.uk (S.R.B.); 2 Wellcome Trust–Medical Research Council Cambridge Stem Cell Institute and Department of Haematology, University of Cambridge, Cambridge CB2 0AW, UK; 3 Section of Cell Biology and Functional Genomics, Imperial College London, London W12 0NN, UK; yusman.manchanda17@imperial.ac.uk (Y.M.); s.bitsi@imperial.ac.uk (S.B.); g.rutter@imperial.ac.uk (G.A.R.); 4 Centre for Bioinformatics and System Biology, Department of Life Sciences, Imperial College London, London SW7 2BX, UK; alessia.david09@imperial.ac.uk; 5 Department of Chemistry, Imperial College London, Molecular Sciences Research Hub, 80 Wood Lane, London W12 0BZ, UK; m.shchepinova@imperial.ac.uk (M.M.S.); e.tate@imperial.ac.uk (E.W.T.); 6 New England Biolabs, Ipswich, MA 01938, USA; correa@neb.com; 7 Institute of Metabolism and Systems Research (IMSR), and Centre of Membrane Proteins and Receptors (COMPARE), University of Birmingham, Birmingham B15 2TT, UK; D.Hodson@bham.ac.uk; 8 Centre for Endocrinology, Diabetes and Metabolism, Birmingham Health Partners, Birmingham B15 2TT, UK; 9 Department of Chemical Biology, Leibniz-Forschungsinstitut für Molekulare Pharmakologie, 13125 Berlin, Germany; broichhagen@fmp-berlin.de; 10 Department of Chemical Biology, Max Planck Institute for Medical Research, 69120 Heidelberg, Germany; 11 Institute of Metabolic Science & MRC Metabolic Diseases Unit, University of Cambridge, Addenbrooke’s Hospital, Hills Road, Cambridge CB2 0QQ, UK; fr222@Cam.ac.uk; 12 Lee Kong Chian School of Medicine, Nanyang Technological University, Singapore 639798, Singapore

**Keywords:** glucagon-like peptide-1, exendin-4, trafficking, biased agonism, degradation, endothelin converting enzyme-1

## Abstract

The glucagon-like peptide-1 receptor (GLP-1R) is an important regulator of blood glucose homeostasis. Ligand-specific differences in membrane trafficking of the GLP-1R influence its signalling properties and therapeutic potential in type 2 diabetes. Here, we have evaluated how different factors combine to control the post-endocytic trafficking of GLP-1R to recycling versus degradative pathways. Experiments were performed in primary islet cells, INS-1 832/3 clonal beta cells and HEK293 cells, using biorthogonal labelling of GLP-1R to determine its localisation and degradation after treatment with GLP-1, exendin-4 and several further GLP-1R agonist peptides. We also characterised the effect of a rare GLP1R coding variant, T149M, and the role of endosomal peptidase endothelin-converting enzyme-1 (ECE-1), in GLP1R trafficking. Our data reveal how treatment with GLP-1 versus exendin-4 is associated with preferential GLP-1R targeting towards a recycling pathway. GLP-1, but not exendin-4, is a substrate for ECE-1, and the resultant propensity to intra-endosomal degradation, in conjunction with differences in binding affinity, contributes to alterations in GLP-1R trafficking behaviours and degradation. The T149M GLP-1R variant shows reduced signalling and internalisation responses, which is likely to be due to disruption of the cytoplasmic region that couples to intracellular effectors. These observations provide insights into how ligand- and genotype-specific factors can influence GLP-1R trafficking.

## 1. Introduction

The glucagon-like peptide-1 receptor (GLP-1R) is a class B G protein-coupled receptor (GPCR) with pleiotropic roles in glucose homeostasis and energy metabolism [1]. Well-characterised effects of GLP-1R activation include augmentation of glucose-stimulated insulin secretion from pancreatic beta cells, suppression of appetite and slowing of gastric emptying. These actions have been harnessed therapeutically for the treatment of type 2 diabetes (T2D) and obesity, with several pharmacokinetically optimised GLP-1R agonists now in routine clinical use [2]. Further applications of GLP-1R agonism have been proposed, including treatment of steatohepatitis [3] and neurodegenerative diseases [4].

Whilst in vitro optimisation of GLP-1R ligands has traditionally focussed on high binding affinity or signalling potency, recent work has highlighted that the balance between recruitment of signal-initiating versus signal-terminating effectors can allow low-affinity “biased” GLP-1R agonists to achieve high therapeutic efficacy [5,6,7,8]. Connected to this, agonist-specific trafficking patterns of the GLP-1R between different endomembrane compartments allow fine-tuning of the duration and spatial origin of intracellular signalling responses [9,10,11,12].

Therapeutic GLP-1R peptide ligands are typically based on the sequence of either the primary endogenous agonist, GLP-1(7-36)-NH_2_, or its homologue exendin-4, a 39 amino acid peptide isolated originally from the venom of the Gila monster [13]. Whilst these are both high-potency full agonists at the GLP-1R, a number of studies have reported differential signal bias and altered trafficking characteristics of these core ligands [11,14,15,16]. In particular, recycling of internalised GLP-1R back to the plasma membrane is slower after exendin-4 treatment, resulting in more pronounced desensitisation in challenge/washout/re-challenge experiments [11,15,17]. The reasons for these differences have not been fully elucidated, but it has been suggested that the trafficking behaviours of GLP-1R ligands may relate to either intra-endosomal ligand-receptor dissociation [6] or to differential ligand sensitivity to intra-endosomal degradation by the endopeptidase endothelin converting enzyme-1 (ECE-1) [18]. Many prior studies have been performed using GLP-1R expressed in heterologous systems and, with increasing recognition that GPCR behaviours can be tissue-specific [19], their findings ideally should be confirmed using beta cell models.

In this study, we have explored the post-endocytic sorting of GLP-1R after treatment with GLP-1 or exendin-4. We focus in particular on ligand-specific differences in receptor degradation, which may in turn contribute to receptor availability and insulin secretory responses during prolonged pharmacological stimulation. We investigate the importance of intra-endosomal ligand proteolysis by ECE-1 through the use of pharmacological inhibition and a panel of chimeric peptide ligands with varying degrees of ECE-1 resistance. We further dissect this phenomenon from the contribution of binding affinity and β-arrestin recruitment by concurrently testing derivatives of these peptides bearing N-terminal substitutions. Finally, we examine the trafficking responses of a natural GLP-1R variant, T149M, previously associated with T2D [20] and known to show impaired intracellular cyclic adenosine monophosphate (cAMP) signalling responses [21].

## 2. Results

### 2.1. GLP-1R Degradation in Beta Cells is More Prominent after Treatment with Exendin-4 versus GLP-1

**Figure 1:**
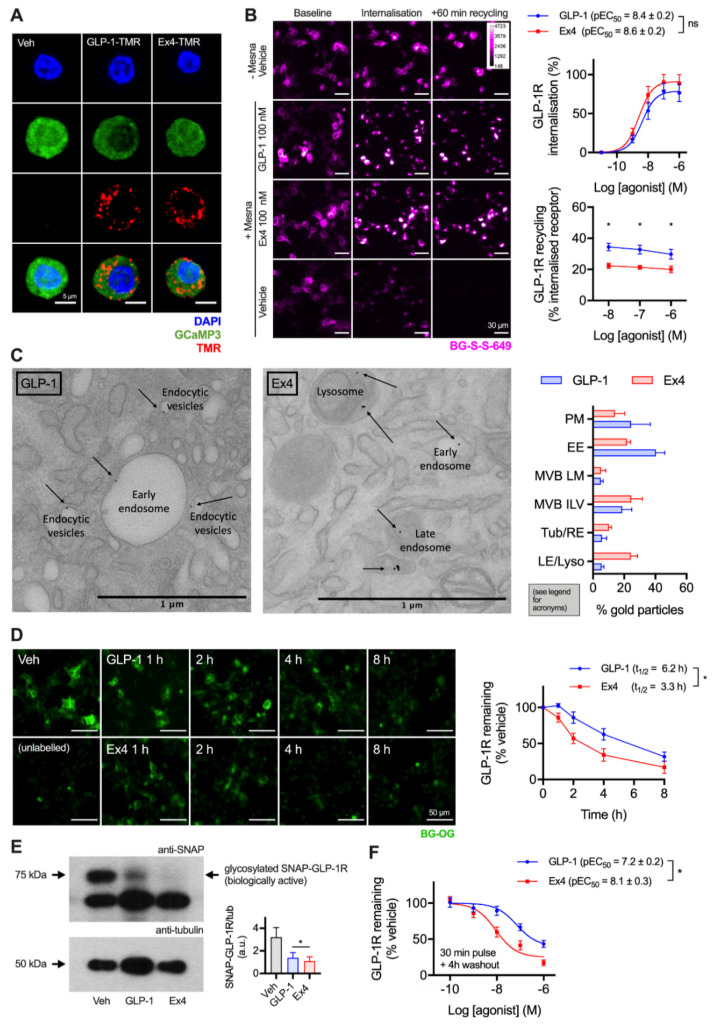
Accelerated GLP-1R degradation with exendin-4 in beta cells. (**A**) Endocytic uptake of 100 nM GLP-1-TMR or exendin-4-TMR in GLP-1R-expressing dispersed primary islet cells. GLP-1R-positive cells are marked by GCaMP3 under GLP-1R promoter-driven Cre expression. Representative images from *n* = 2 mice. Scale bar = 6 µm. The sequences and pharmacological characterisation of GLP-1-TMR and exendin-4-TMR are provided in the Supporting Information (Supplementary Figure S1). (**B**) Assessment of GLP-1R trafficking in INS-1-SNAP-GLP-1R cells using high content microscopy. Representative images and quantification of GLP-1R internalisation (30 min) and recycling (60 min) from *n* = 5 experiments is shown, with internalisation pEC_50_ compared by paired *t*-test, and recycling results compared by two-way randomised block ANOVA with Sidak’s test. Scale bar = 30 µm. (**C**) Example electron micrographs in MIN6B1-SNAP-GLP-1R cells labelled with BG-S-S-biotin and streptavidin-gold (10 nm) and treated with 100 nM exendin-4 and GLP-1 for 1 h. Quantification of gold particle distribution from *n* = 3 experiments comprising plasma membrane (PM), early endosomes (EE), multivesicular body limiting membrane (MVB LM) or intraluminal vesicles (ILV), recycling endosomes or tubules (Tub/RE) and late endosomes or lysosomes (LE/Lyso). Scale bar = 1 µm. (**D**) Time-course for GLP-1R degradation in INS-1-SNAP-GLP-1R cells treated with cycloheximide (50 µg/mL) in serum-free medium ± 10 nM agonist, applied in reverse time order, followed by BG-OG labelling (0.5 µM) for the final 30 min of the study. Representative images and quantification from *n* = 4 experiments are shown, with t_1/2_ quantified by 1-phase exponential curve fitting and compared by paired *t*-test. Scale bar = 50 µm. (**E**) Western blot analysis and quantification of GLP-1R degradation in INS-1-SNAP-GLP-1R cells after treatment for 6 h in serum-free medium supplemented with 50 µg/mL cycloheximide in the presence or absence of 10 nM GLP-1 or exendin-4, quantification from *n* = 4 experiments and comparison by paired *t*-test. (**F**) GLP-1R degradation in INS-1-SNAP-GLP-1R cells pre-treated with cycloheximide (50 µg/mL) in serum-free medium for 60 min before application of agonist for 30 min, followed by washout and 4 h incubation; 3-parameter fit of data from *n* = 5 experiments. * *p* < 0.05 by statistical test indicated. Data represented as mean ± SEM.

GLP-1R is typically described as a fast-internalising receptor [22]. To provide evidence that this is the case for endogenous GLP-1R in its native cellular context, we first visualised uptake of fluorescent GLP-1 and exendin-4 conjugates (see Supplementary Figure S1) in dispersed primary islet cells, with GLP-1R expression marked by the fluorescent reporter GCaMP3 under Cre-dependent control [23] (Figure 1A). Both ligands were extensively taken up into punctate endosome-like structures. As agonist and receptor may dissociate on entering the endocytic pathway, to study the post-endocytic trafficking of GLP-1R in a beta cell context we used cultured INS-1 832/3 cells stably expressing N-terminally SNAP-tagged GLP-1R (referred to hereafter as INS-1-SNAP-GLP-1R cells) [24]. A cleavable benzyl guanine (BG)-conjugated fluorescent probe, BG-S-S-649, allowed quantification of internalised and subsequently recycled SNAP-GLP-1R by high content microscopy, as previously described [11]. Pronounced GLP-1R internalisation was observed with both ligands at high concentrations, with exendin-4 showing slightly increased potency (Figure 1B). Moreover, following agonist washout, GLP-1R recycled more slowly after exendin-4 treatment over a wide concentration range. We also visualised post-endocytic sorting of SNAP-GLP-1R by electron microscopy after treatment with either ligand in MIN6 mouse beta cells, with the receptor surface-labelled using BG-biotin and streptavidin-gold prior to treatment. This indicated a trend towards greater localisation of GLP-1R in late endosomes and lysosomes after exendin-4 treatment compared to GLP-1 (Figure 1C).

To examine the consequences of these apparent differences in post-endocytic sorting on GLP-1R degradation, experiments were performed in INS-1-SNAP-GLP-1R cells treated with cycloheximide to arrest protein translation. After continuous exposure to 10 nM of either ligand, applied in a time series in reverse order, a cell permeating SNAP-tag probe, BG-conjugated Oregon Green (BG-OG) [25], was used to label total residual intact SNAP-GLP-1R. Over an 8 h time-course, a steady reduction of BG-OG labelling was apparent with both ligands, suggesting destruction of the SNAP moiety (and by inference, the GLP-1R itself), but this was faster and more pronounced with exendin-4 treatment (Figure 1D). Note that BG-OG labelling of internalised SNAP-GLP-1R after 30 min treatment with 100 nM agonist was similar for both agonists, suggesting that the fluorescent signal reduction seen in Figure 1D did not represent the effect of endosomal pH on fluorophore emission intensity per se (Supplementary Figure S2). Moreover, densitometric Western blotting analysis following 6 h stimulation with 10 nM agonists in INS-1-SNAP-GLP-1R cells confirmed greater SNAP-GLP-1R degradation with exendin-4 (Figure 1E). Additionally, 4 h after a 30 min pulsed agonist treatment and washout, loss of GLP-1R signal was more pronounced throughout the concentration range tested with exendin-4 compared to GLP-1 (Figure 1F). Because residual extracellular agonist was removed in the latter experiment, the result is likely to represent the differential effects of internalised GLP-1R-exendin-4 versus -GLP-1 complexes on post-endocytic targeting.

### 2.2. GLP-1R Degradation Rates are Associated with Ligand Sensitivity to ECE-1

**Figure 2:**
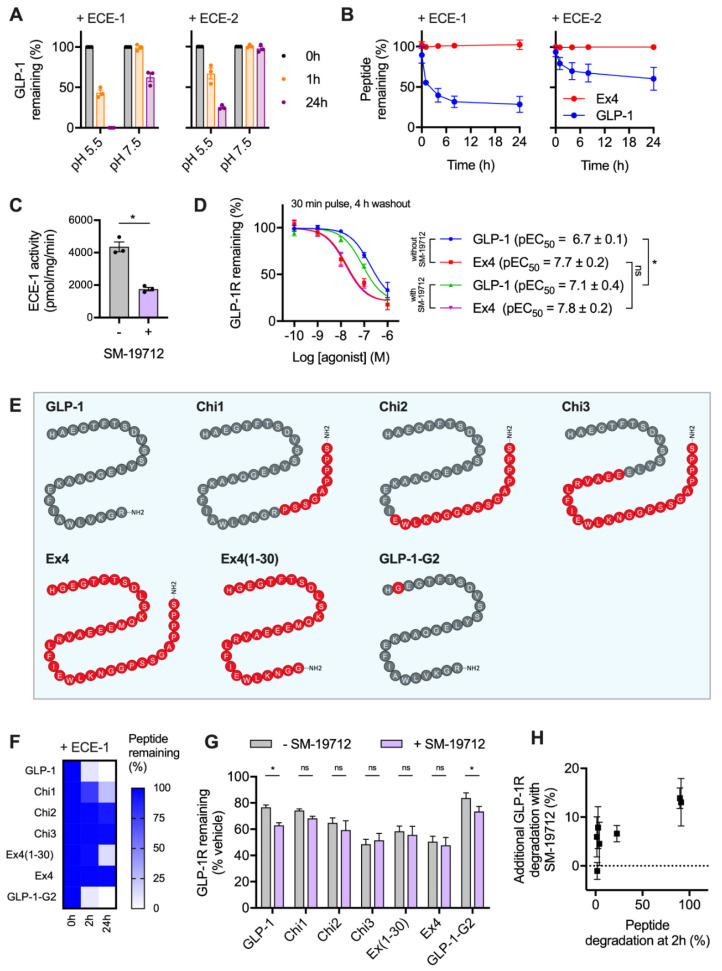
GLP-1R degradation is partly influenced by ECE-1-mediated ligand proteolysis. (**A**) In vitro GLP-1 degradation at indicated pH in the presence of 0.25 µg ECE-1 or ECE-2, determined by HPLC quantification from *n* = 3 experiments. (**B**) Time-course of in vitro GLP-1 versus exendin-4 degradation by ECE-1 or ECE-2 at pH 5.5, quantified from *n* = 2 experiments by HPLC. (**C**) ECE-1 activity measured from INS-1-SNAP-GLP-1R cell lysates ± 10 µM SM-19712, *n* = 3, compared by paired *t*-test. (**D**) GLP-1R degradation in INS-1-SNAP-GLP-1R cells pre-treated with cycloheximide (50 µg/mL) in serum-free medium for 60 min before application of agonist for 30 min, followed by washout and 4 h incubation with serum-free medium containing cycloheximide ± 10 µM SM-19712; 3-parameter fit of data from *n* = 5 experiments, with pEC_50_ compared by 2-way randomised block ANOVA with Sidak’s test. (**E**) Schematic showing amino acid sequences of chimeric peptides, with GLP-1-derived sequences in grey and exendin-4-derived sequences in red. (**F**) Time-course of in vitro degradation of chimeric peptides by ECE-1 at pH 5.5, quantified from *n* = 3 experiments by HPLC. (**G**) GLP-1R degradation in INS-1-SNAP-GLP-1R cells pre-treated with cycloheximide (50 µg/mL) in serum-free medium for 60 min before application of 100 nM chimeric peptide for 30 min, followed by washout and 4 h incubation with serum-free medium containing cycloheximide ± 10 µM SM-19712, *n* = 5, compared by 2-way randomised block ANOVA with Sidak’s test. (**H**) Analysis of interaction between ECE-1 sensitivity as indicated in (**F**) and additional GLP-1R degradation ± SM-19712 treatment as shown in (**G**). * *p* < 0.05 by statistical test indicated. Data represented as mean ± SEM.

It was recently reported that GLP-1R trafficking in HEK293 cells is regulated by the activity of ECE-1 [18], a pH-sensitive endopeptidase known to degrade various GPCR peptide ligands once they enter the endocytic system. The resultant dissociation of the ligand-receptor complex typically prompts preferential targeting of the receptor to a recycling pathway [26,27]. Due to the known differences in proteolytic stability of GLP-1 versus exendin-4 to the related neutral endopeptidase NEP24.11 [28], we aimed to experimentally verify whether ECE-1 sensitivity might underpin the observed differences in post-endocytic sorting with these two ligands in beta cells. We first performed in vitro digestion assays of GLP-1 using ECE-1 and ECE-2 under neutral and acidic pH conditions, to mimic the extracellular and intra-endosomal environments, respectively. Here, high performance liuid chromatography (HPLC) measurements indicated extensive peptide degradation, particularly by ECE-1 at low pH (Figure 2A). Exendin-4 was highly resistant to ECE-1 and ECE-2 degradation, remaining entirely intact after a 24 h incubation with either enzyme at low pH (Figure 2B).

To determine the importance of ECE-1-mediated agonist degradation on post-endocytic GLP-1R sorting, we used the specific ECE-1 inhibitor SM-19712 [18,26,29], which inhibited ECE-1 activity in INS-1-SNAP-GLP-1R cells by approximately 60% when applied at 10 µM (Figure 2C). Under these conditions, reduced degradation of ECE-1-sensitive GLP-1 is expected to result in greater targeting of GLP-1R towards a degradative pathway, without affecting responses to ECE-1-resistant exendin-4. Indeed, treatment with SM-19712 for 4 h after exposure to GLP-1 led to a two-fold increase in potency for GLP-1R degradation, without affecting the response to exendin-4 (Figure 2D). However, even in the presence of SM-19712, exendin-4 remained five-fold more potent for GLP-1R degradation, suggesting that ECE-1 sensitivity is unlikely to be the only reason for the differential GLP-1R degradative potential of these two ligands.

We also performed further studies using a series of chimeric peptides sharing features of GLP-1 and exendin-4 (Figure 2E), which we recently reported to show divergent effects on GLP-1R recycling [11]. In the present study, we measured breakdown of each of these ligands by ECE-1 in acidic conditions (Figure 2F), finding that certain exendin-4-like features are important in conferring resistance to ECE-mediated proteolysis. Of note, removal of the exendin-4 C-terminal “Trp-cage” to generate exendin-4(1-30) led to a partial reduction in ECE-1 resistance, as previously noted with NEP24.11 [30]. Correspondingly, addition of the Trp-cage to the C-terminus of GLP-1 (generating “Chi1”) resulted in a partial increase in ECE-1 resistance. As expected, substitution of A→G at position two of GLP-1, which confers resistance to the exopeptidase dipeptidyl dipeptidase-4 (DPP-4), had no effect on ECE-1 sensitivity.

Using these ligands, we observed variable degrees of GLP-1R degradation in INS-1-SNAP-GLP-1R cells 4 h after pulsed treatment. ECE-1-sensitive peptides (GLP-1, GLP-1-G2, Chi1) tended to result in less GLP-1R degradation than did the most ECE-1-resistant agonists (exendin-4, Chi2, Chi3) (Figure 2G). Moreover, the relative ability of ECE-1 inhibition using SM-19712 to increase GLP-1R degradation was somewhat more marked for the ECE-1-sensitive peptides, with the relationship between these two factors depicted in Figure 2H.

These findings provide further support for a role, albeit a partial one, of ECE-1 in control of post-endocytic targeting of GLP-1R in beta cells.

### 2.3. Peptide Agonist N-Terminal Substitutions Influence GLP-1R Degradation

Ligand binding affinity is a further factor that influences post-endocytic receptor sorting, achieved by controlling the stability of internalised ligand-receptor complexes [6,11,31]. Our earlier work has highlighted how a His → Phe amino acid substitution at position 1, and a Glu → Asp switch as position 3, tend to oppositely influence a number of pharmacological parameters of GLP-1R peptide ligands, including binding affinity, signal bias, GLP-1R palmitoylation and lipid raft clustering [6,11,24]. Thus, asp3 and phe1 derivatives of the chimeric peptide panel in Figure 2 provide means to probe the role of further factors beyond proteolytic stability in the control of ligand-induced GLP-1R degradation. We have performed a prior evaluation of some of the phe1 ligands in an earlier work [11], using different experimental conditions and analytical approaches.

**Figure 3:**
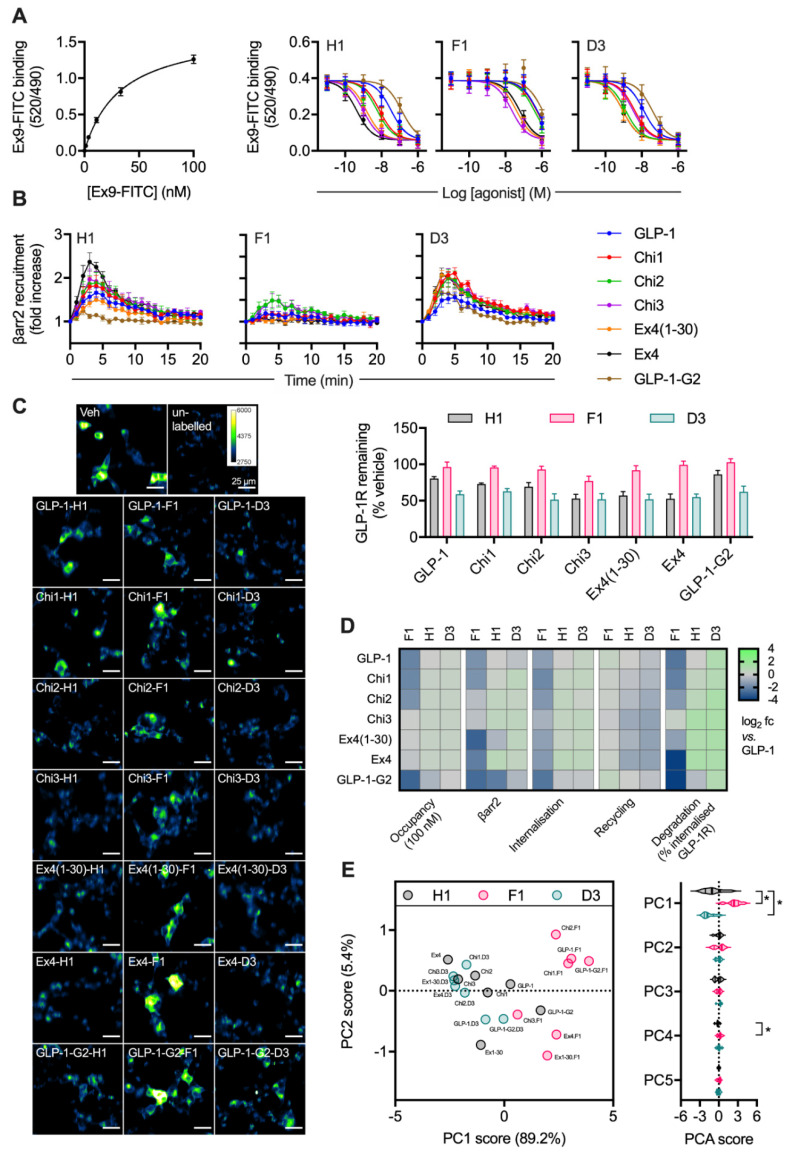
Relationship between binding affinity and GLP-1R degradation rate with N-terminally substituted GLP-1R agonists. (**A**) TR-FRET equilibrium binding affinity assays performed in HEK293-SNAP-GLP-1R cells, with saturation binding of exendin(9-39)-FITC (“Ex9-FITC”) and effect of indicated unlabelled agonist on binding of 10 nM exendin(9-39)-FITC in parallel experiments, *n* = 6. (**B**) β-arrestin-2 recruitment to GLP-1R in HEK293T cells, measured using nanoBiT complementation, with 100 nM of indicated agonist, *n* = 4. (**C**) GLP-1R degradation in INS-1-SNAP-GLP-1R cells pre-treated with cycloheximide (50 µg/mL) in serum-free medium for 60 min before application of 100 nM peptide for 30 min, followed by washout and 4 h incubation with serum-free medium containing cycloheximide, *n* = 6, with representative images provided. (**D**) Heatmap summarising the pharmacological properties of each agonist at 100 nM, displayed as log_2_ fold difference after normalisation to GLP-1 response. Occupancy has been inferred from the agonist K_i_ using the law of mass action. (**E**) Principal component analysis of -his1, -phe1 and -asp3 ligands, with 2D plot showing PC1 versus PC2 with loadings indicated on the axes, and violin plot indicating PC1-5 with comparison within each component by randomised block one-way ANOVA with Dunnett’s test versus -his1 ligand. * *p* < 0.05 by statistical test indicated. Data represented as mean ± SEM.

Competition binding experiments in HEK293-SNAP-GLP-1R cells demonstrated a wide range of binding affinities for this ligand panel (Figure 3A, Table 1). The phe1 substitution tended to decrease affinity, whereas the asp3 modification had the opposite effect in some (but not all) cases. Efficacy for β-arrestin-2 recruitment, a parameter commonly associated with GPCR endocytosis, was assessed in HEK293 cells using nanoBiT complementation [7,32], with phe1 ligands showing attenuated responses, as expected (Figure 3B, Table 1). GLP-1R endocytosis and recycling were assessed in INS-1-SNAP-GLP-1R cells, as was GLP-1R degradation after pulsed agonist treatment and washout (Figure 3C, Table 1). Treatment with phe1 ligands, in keeping with the well-established trafficking pattern typically seen with this N-terminal substitution, was characterised by slow internalisation, rapid recycling and a lower degree of GLP-1R degradation. Asp3 ligands tended to lead to somewhat reduced GLP-1R recycling, as well as to increased degradation with the more GLP-1-like peptides. The pharmacological effects of the full set of ligands are also summarised by heatmap representation in Figure 3D, in which the degradation result has also been normalised to the previously determined internalisation results to allow easier visual comparison of GLP-1R targeting towards recycling versus degradation. Finally, using principal component analysis to identify ligand similarity after dimensionality reduction from multiple pharmacological metrics, it was apparent that phe1 ligands were most dissimilar to the others, with asp3 peptides also differentiated from his1 equivalents in principal component 1 (PC1) (Figure 3E).

As the phe1 and asp3 substitutions are distant from the putative endopeptidase target zone, these findings highlight how further ligand factors beyond ECE-1 susceptibility influence the degradation of internalised GLP-1R.

### 2.4. A Natural GLP-1R Variant Shows Altered Trafficking Response

**Figure 4:**
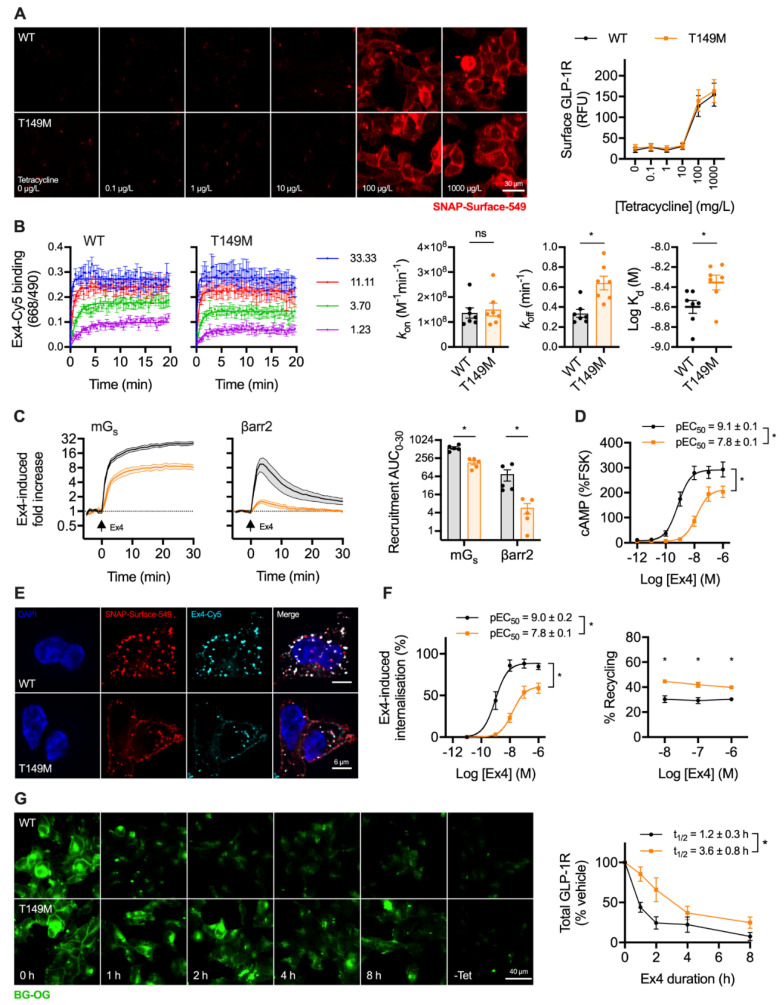
Trafficking and signalling with the T149M GLP-1R variant. (**A**) Tetracycline-induced expression of wild-type and T149M SNAP-GLP-1R in stably transfected Flp-In 293-T-REx cells, with quantification from *n* = 5 experiments; scale bar = 30 µm. (**B**) Assessment of kinetic binding parameters of exendin-4-C39-Cy5 in 293-T-Rex-GLP-1R cells, *n* = 7, with comparisons by paired *t*-test. (**C**) Recruitment of mini-G_s_ (mG_s_) and β-arrestin-2 (βarr2) in response to 100 nM exendin-4, assessed by nanoBiT complementation, *n* = 5, with AUC quantification and comparison by paired *t*-test. (**D**) cAMP responses to exendin-4 in 293-T-REx-GLP-1R cells, normalised to 10 µM forskolin (FSK), *n* = 5, with pEC_50_ and E_max_ compared by paired *t*-tests. (**E**) GLP-1R internalisation and uptake of exendin-4-C39-Cy5 uptake in 293-T-REx-GLP-1R stimulated for 30 min, representative images of *n* = 3 experiments; scale bars = 6 µm. (**F**) Quantification of GLP-1R internalisation and recycling by high content microscopy in 293-T-REx-GLP-1R cells, *n* = 5, with internalisation pEC_50_ and E_max_ compared by paired *t*-tests, and recycling results compared by two-way randomised block ANOVA with Sidak’s test. (**G**) GLP-1R degradation in 293-T-REx-GLP-1R cells stimulated with 100 nM exendin-4 and subsequently labelled with BG-OG (0.5 µM), with representative images and quantification from *n* = 5 experiments, with t_1/2_ compared by paired *t*-test. * *p* < 0.05 by statistical test indicated. Data represented as mean ± SEM.

GLP-1R-T149M is a rare GLP-1R single nucleotide coding variant (rs367543060) originally identified in a T2D patient cohort [20]. Impaired signalling responses have been described for this variant [21,33], as well as reduced internalisation in response to GLP-1 [34]. We used the Flp-In T-REx system to achieve isogenic, tetracycline-inducible expression of wild-type and T149M variant GLP-1R, with both receptor types SNAP-tagged at the N-terminus and bearing a SmBiT tag at the C-terminus. Surface localisation of each receptor type after tetracycline induction was similar (Figure 4A), consistent with earlier reports [33,34].

Exendin-4 coupled at the C-terminus to the far-red fluorophore Cy5 using cysteine-maleimide conjugation (see Supplementary Figure S1), as previously performed using the antagonist ligand exendin(9-39) [35], was used to evaluate ligand binding kinetics. GLP-1R-T149M showed a modestly increased dissociation rate, which translated to a small reduction in affinity (Figure 4B). However, there was a dramatic loss in exendin-4-induced recruitment of mini-G_s_ [36] and β-arrestin-2 [32] with the T149M variant, as assessed by nanoBiT complementation (Figure 4C), suggesting a pronounced signalling deficit. Indeed, potency for cAMP production was reduced 20-fold, with efficacy also reduced (Figure 4D). GLP-1R internalisation, visualised in parallel with exendin-4-Cy5 endocytic uptake, was reduced with T149M (Figure 4E). Endocytosis and recycling were further quantified by high content microscopy, which showed that maximum internalisation was reduced by approximately 50%, accompanied by a potency reduction comparable to that observed for cAMP production (Figure 4F). Correspondingly, GLP-1R recycling was modestly increased. GLP-1R degradation by prolonged incubation with exendin-4 was assessed after first washing out tetracycline to halt ongoing transcription of GLP-1R. In keeping with the results of the prior trafficking assay, this indicated delayed degradation of T149M compared to wild-type over an 8 h time-course (Figure 4G).

**Figure 5:**
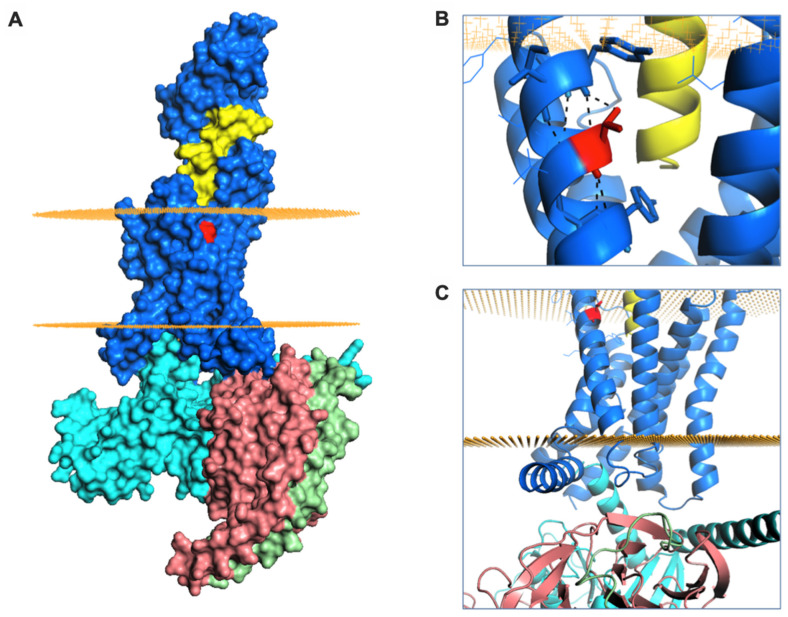
GLP-1R structure and T149 structural role. (**A**) Surface representation of GLP-1R (in blue) bound to exendin-P5 (in yellow) and guanine nucleotide-binding protein G_s_ subunit α (in cyan), β (in salmon) and γ (in green). The intracellular and extracellular boundaries of the lipid bilayer are presented as orange disks. T149 is coloured in red. (**B**) The intrahelical hydrogen bond formed by T149 (in red) is highlighted with black dashes. (**C**) Cartoon representation of GLP-1R structure highlighting the juxta-membrane and intracellular region of TM1. The G protein subunits are also shown.

Finally, we aimed to better understand the signalling and trafficking defect observed with GLP-1R T149M by analysis of GLP-1R structures described in the literature. As the exendin-4-bound GLP-1R structure has not been reported, we examined two full-length GLP-1R active structures bound by different orthosteric agonists (exendin-P5 and GLP-1), both complexed with the G_s_ protein heterotrimer [37,38]. T149 is a highly conserved residue located in the first transmembrane helix (TM1) of GLP-1R (Figure 5A). The polar T149 residue is positioned towards the extracellular side of the membrane and stabilizes the tilt of the TM1 helix by establishing a network of intrahelical hydrogen bonds (Figure 5B, Supplementary Figure S3A). Although not directly involved in binding to exendin-P5 or GLP-1, T149 is located in close proximity to the ligand binding pocket (Figure 5A, Supplementary Figure S3A). Substitution of the polar threonine with the hydrophobic methionine is predicted to affect GLP-1R conformation via disruption of the intrahelical hydrogen bond network. This is likely to result in a change in the TM1 tilt, with possible displacement of residues located towards the intracellular side of the membrane, where interaction with G proteins occurs (Figure 5C) [39], a prediction that is consistent with our observation of reduced mini-G_s_ coupling with GLP-1R-T149M. Whilst the position of β-arrestin complexed to GLP-1R has not been reported, structural analysis of another GPCR-β-arrestin complex [40] suggests that the intracellular loop 1 (ICL1) of GLP-1R is likely to be involved in a complex network of physico-chemical interactions between GLP-1R and β-arrestin (Supplementary Figure S3B). It can therefore be hypothesized that the conformational changes induced by T149M may affect correct coupling of these two proteins to GLP-1R. In addition, introduction of the large hydrophobic side chain of methionine results in a more favourable GLP-1R interaction with the lipid bilayer, thus providing additional driving forces for TM1 helix tilting, as observed for other GPCRs [41]. Introduction of this large hydrophobic residue results in increased thermostability, with an overall stabilizing effect on GLP-1R protein (free energy change ΔΔG + 3 kcal/mol, confirmed with both experimental GLP-1R structures analysed). This is likely to reduce GLP-1R intrinsic flexibility [42], thereby resisting transition to an active receptor conformation on agonist binding.

## 3. Discussion

**Figure 6:**
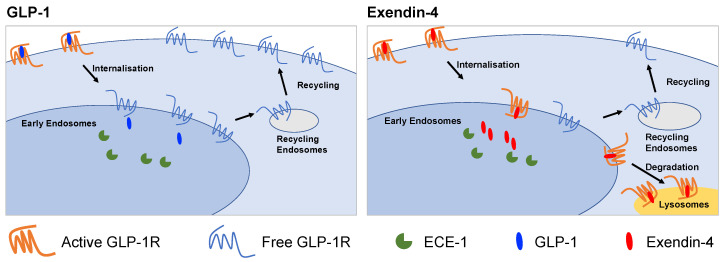
Graphical summary of differences between GLP-1R trafficking induced by GLP-1 and exendin-4. GLP-1 favours GLP-1R recycling to the plasma membrane whereas exendin-4 promotes lyosomal targeting. The action of ECE-1 and binding affinity are two factors that influence this process.

In this study, we have examined factors that influence the post-endocytic fate of GLP-1R when activated by two prototypical agonists for this receptor: GLP-1 and exendin-4. After first demonstrating the effect of each agonist on GLP-1R recycling and degradation in a pancreatic beta cell context, we tested how these parameters are linked to resistance to proteolytic degradation, binding affinity and recruitment of intracellular effectors. We also performed studies using a natural GLP-1R variant, T149M, which showed attenuated signalling and internalisation responses to exendin-4, but faster recycling and slower degradation. The key findings from our study are summarized in Figure 6.

Increasing interest in how dynamic changes in GLP-1R subcellular localisation influence its ability to promote downstream responses, such as insulin release, provides the context to this work. Like many other GPCRs [43], entry into the endocytic pathway may be required for a full GLP-1R intracellular signalling response [9,44]. However, with prolonged pharmacological stimulations, mounting evidence from studies using biased agonists with distinctive GLP-1R trafficking characteristics [6,7,11,16,45] suggests that preservation of a minimum level of surface-resident GLP-1R becomes a limiting factor for insulin secretion. A fuller understanding of the range of factors influencing these processes is needed so they can be optimally leveraged for therapeutic benefit.

Detailed examination of GLP-1R trafficking is frequently conducted in non-beta cells [15,18], and our work using beta cell models presents an environment more akin to that encountered by GLP-1R in its native context. Here, we used primary islet cells to confirm extensive endocytic uptake of both GLP-1 and exendin-4 in a “gold standard” model. However, as biorthogonal labelling of GLP-1R in primary cells is currently not feasible, further studies to investigate the post-endocytic behaviours of GLP-1R were performed using SNAP-tagged GLP-1R expressed in clonal beta cells. As expected, both GLP-1 and exendin-4 induced pronounced GLP-1R internalisation, but with differences apparent in subsequent recycling to the plasma membrane versus lysosomal degradation. To understand the processes underpinning this divergence, we first built on recent work highlighting a potential role for the endopeptidase ECE-1 in the control of GLP-1R trafficking [18], as previously shown for other GPCRs [26,46]. We confirmed that GLP-1 (but not exendin-4) is a substrate for this enzyme at acidic pH—a prerequisite for regulation of GLP-1R trafficking by endosomal ECE-1. We also demonstrated that GLP-1-induced GLP-1R degradation in beta cells is modestly increased by ECE-1 inhibition. However, this effect did not entirely explain the marked difference between GLP-1R degradation rates observed with GLP-1 versus exendin-4, which might be due to the incomplete inhibition achieved in our studies, reflect the activity of other endosomal endopeptidases not inhibited by SM-19712, or involve further mechanisms unrelated to endopeptidase degradation of the ligand. Nevertheless, the potential for therapeutic strategies based on ECE-1 inhibition [47] to affect the function of GLP-1R and other peptide GPCRs as an unintended consequence should be borne in mind. It could further be envisaged that GLP-1R ligands engineered for reduced resistance to ECE-1 might enhance plasma membrane GLP-1R availability, thereby allowing longer-lasting insulinotropic responses through faster recycling. Designing such a peptide whilst maintaining an adequate degree of protection against extracellular endopeptidases is, however, likely to be challenging.

Biased agonism can be leveraged to improve the therapeutic targeting of GPCRs, including GLP-1R [5,48,49]. Here, we used biased GLP-1R ligands with N-terminal substitutions as tool compounds to unpick the relative contributions of proteolytic stability from other pharmacological parameters that control agonist-mediated GLP-1R degradation. From these studies, it was clear, as expected, that many factors beyond resistance to ECE-1 influence how internalised GLP-1R agonists can control the downstream fate of the receptor. Peptide variants with a phe1 or asp3 substitution typically showed trafficking bias towards, respectively, recycling or degradation of the GLP-1R, despite the sequence change being distant from the likely cleavage site for ECE-1. These differences were frequently related to GLP-1R binding affinity, with high-affinity ligands showing a preference for GLP-1R degradation. Differences in persistence of ligand-receptor complexes within the endosome are likely to explain this association [50]. This rule did not universally apply; for example, exendin-phe1, a biased ligand with promising anti-diabetic characteristics in vivo such as enhanced glucose lowering and reduced nausea compared to exendin-4 [6], exhibited similar binding affinity to GLP-1 but yielded considerably less GLP-1R degradation, even after differences in GLP-1R internalisation were taken into account. GLP-1R degradation rates were also generally lower than predicted for asp3 substitutions in exendin-4 and exendin-4-like peptides, opening the possibility, as previously shown for other receptors [51,52,53], of the existence of alternative intracellular trafficking or sequestration behaviours of the GLP-1R, e.g., to specialised non-degradative endosomal subpopulations. Overall, this series of peptide agonists is a useful resource for the study GLP-1R trafficking, and could be used, for example, to probe correlation of trafficking phenotypes with quantitative analyses of ligand-induced interactions made by the receptor with candidate trafficking effectors.

Large population genomic studies have provided evidence of significant natural genomic variability in GPCR coding sequences, which may partly underpin inter-individual responses to therapeutic GPCR ligands [54]. In the present work, we studied the GLP-1R variant T149M, which is a candidate risk allele for T2D [20], albeit with confirmation awaited from larger studies. Pharmacological evaluations of this variant have consistently highlighted a pronounced signalling defect which does not appear to result primarily from reduced expression or ligand binding [33,34,55]. In our hands, GLP-1R T149M showed profoundly reduced recruitment of both mini-G_s_ and β-arrestin-2, with the latter particularly attenuated. This pharmacological observation can be explained by the detrimental effect of the T149M substitution on TM1 tilt and resultant disruption of the cytoplasmic regions required for efficient coupling to G protein heterotrimers and β-arrestins. Moreover, GLP-1R internalisation was also markedly reduced, although still detectable. The latter contrasts somewhat with a previous study [34] in which no internalisation of the T149M GLP-1R variant could be observed with GLP-1 treatment, with this discrepancy probably reflecting differences in the ligands used in both studies. We found that GLP-1R T149M recycles faster post exendin-4-induced internalisation, with an associated slower rate of GLP-1R degradation. Interestingly, the combination of reduced β-arrestin-2 recruitment efficacy, slower GLP-1R endocytosis but faster recycling and slower target receptor degradation, recapitulates the pharmacological profile of stereotypical G protein-biased GLP-1R agonists such as exendin-phe1 which, paradoxically, show increased insulinotropic efficacy. In the future, it may be valuable to study this and other receptor variants in a beta cell environment to determine how coupling efficiencies translate to insulin release.

Our study has a number of limitations. Firstly, experiments were in the most part conducted in isolated clonal beta cells, which may not fully model the behaviour of fully differentiated primary beta cells. Whilst we did make use of fluorescent ligands to confirm the basic endocytic profile of exendin-4 and GLP-1, following the agonist-directed intracellular journey of GLP-1R in a manner similar to our use of SNAP-GLP-1R surface labelling is not easily achieved in primary beta cells, as specific GLP-1R antibodies capable of labelling the extracellular receptor face compete with agonists for the orthosteric binding pocket [56] and are therefore not suitable for these assays. Moreover, cell populations within the pancreatic islet are highly interconnected [57,58], and we did not perform comparative ligand uptake studies with intact isolated islets or in vivo, as previously performed with fluorophore-conjugated exendin-4 [15]. Our investigation into the role of ECE-1 was partially hampered by the fact that we achieved only partial ECE-1 inhibition using a standard dose of the small molecule inhibitor SM-19712. To avoid non-specific off-target effects from higher inhibitor concentrations, genetic approaches such as CRISPR-Cas9 could be used to knock-out ECE-1 in clonal beta cells, or beta cell-specific ECE-1 knockout animals could be established to confirm whether this effect is physiologically important. Finally, we included a single, rare GLP-1R variant in this report, with a reported minor allele frequency of 1 × 10^−5^ [59]. Higher prevalence GLP-1R variants should be evaluated to provide a more comprehensive picture of how genomic variation could impact on responses to therapeutic GLP-1R agonists [54]. From the structural point of view, caveats apply to our models and interpretation as no full-length exendin-4-bound GLP-1R structure is available, requiring us to rely on structural models with alternative (albeit similar) orthosteric ligands bound.

The distinct trafficking phenotypes of GLP-1 versus exendin-4 are of potential clinical importance as approved GLP-1R agonist treatments are essentially derivatives of these two peptides. Liraglutide, Semaglutide, Dulaglutide and Albiglutide share a high degree of homology with GLP-1, whereas Exenatide and Lixisenatide are, respectively, identical or highly similar to exendin-4. It might be expected that these ligands will show differences in their trafficking phenotypes, and indeed this appears to be the case [6]. However, the impact of the conjugated acyl chain (Liraglutide, Semaglutide), Fc fragment (Dulaglutide) or albumin moiety (Albiglutide) beyond that of the peptide itself is not clear, with recent work highlighting how acylation can protect GLP-1R agonists from degradation by NEP24.11 [30].

In summary, this work demonstrates how ligand-, cell- and receptor-specific factors can influence the post-endocytic sorting of GLP-1R, providing an opportunity to optimise the therapeutic potential of this treatment class.

## 4. Materials and Methods

### 4.1. Reagents

Custom peptides were purchased from Wuxi Apptec (Shanghai, China) and were at least 90% pure. Synthesis of exendin-4-Cy5 is described in Supporting Information (Supplementary Methods). SNAP-Surface probes were purchased from New England Biolabs (Ipswich, MA, USA). BG-S-S-649 [11] and BG-S-S-biotin [6] were provided by New England Biolabs on a collaborative basis. BG-OG has been previously described [24]. The source of other reagents is described in the relevant sections.

### 4.2. Plasmids

pSNAP-GLP-1R was obtained from Cisbio (Codolet, France). FLAG-GLP-1R-SmBiT was generated as previously described [7]. The plasmid for mini-G_s_ carrying an N-terminal LgBiT tag [35] was a gift from Prof Nevin Lambert, Medical College of Georgia. The plasmid for β-arrestin-2 fused at the N-terminus to LgBiT (plasmid CS1603B118) was obtained from Promega custom assay services (Madison, WI, USA). Custom GLP-1R expression plasmids, wild-type and T149M mutant pcDNA5-SNAP_f_-GLP-1R-SmBiT, were generated by Genewiz (Leipzig, Germany) to the following design: codon-optimised wild-type or T149M variant human GLP-1R (lacking the endogenous N-terminal signal peptide) was tagged at the N-terminus with a fast-labelling SNAP_f_ tag and upstream signal peptide based on that of the 5-HT_3A_ receptor (MDSYLLMWGLLTFIMVPGCQA), plus C-terminal SmBiT tag, and inserted into the pcDNA5/FRT/TO expression vector. pOG44 was obtained from Thermo Fisher (Waltham, MA, USA).

### 4.3. Cell Culture

INS-1-SNAP-GLP-1R cells, generated by stably expressing pSNAP-GLP-1R in INS-1 832/3 cells lacking endogenous GLP-1R expression after deletion by CRISPR/Cas9 (a gift from Dr Jacqueline Naylor, AstraZeneca) [60], have been previously described [23] and were maintained in RPMI-1640 with 11 mM glucose, 10 mM HEPES, 2 mM glutamine, 1 mM sodium pyruvate, 50 µM β-mercaptoethanol, 10% foetal bovine serum (FBS), 1% penicillin/streptomycin and 1 mg/mL G418. MIN6B1-SNAP-GLP-1R cells [10], derived from the parental MIN6B1 subline [61] (a gift from Prof Philippe Halban, University of Geneva), were maintained in DMEM supplemented with 50 µM β-mercaptoethanol, 15% FBS, 1% penicillin/streptomycin and 1 mg/mL G418. HEK293T and Flp-In™ T-REx™ 293 cells (Thermo Fisher) were maintained in DMEM supplemented with 10% FBS and 1% penicillin/streptomycin. To obtain populations of isogenic SNAP-GLP-1R-SmBiT and SNAP-GLP-1R-T149M-SmBiT expressing cells, T-REx-293 cells were co-transfected with pOG44 and pcDNA5-SNAP_f_-GLP-1R-SmBiT or SNAP_f_-GLP-1R T149M-SmBiT in a 9:1 ratio, followed by selection with 100 µg/mL hygromycin.

### 4.4. Fluorescence Imaging of Fixed Samples

Cells grown on poly-D-lysine-coated coverslips were fixed with 4% paraformaldehyde for 15 min at room temperature, washed, and mounted onto slides in Diamond Prolong anti-fade with DAPI (Thermo Fisher). Imaging was performed using a Nikon Ti2-E microscope controlled by custom scripts [62] implemented in µManager [63], with an LED light source (CoolLED). Z-stacks were acquired at 100× using a 1.45 numerical aperture oil immersion objective and were deconvolved with DeconvolutionLab2 [64] in Fiji (v1.53a) using the Richard-Lucy algorithm.

### 4.5. Electron Microscopy

MIN6B1-SNAP-GLP-1R cells were seeded onto Thermanox coverslips (Agar Scientific, Essex, UK), labelled with 2 μM cleavable BG-S-S-biotin surface SNAP-tag probe at 37 °C for 30 min and secondarily labelled with 5 μg/mL AlexaFluor 488 Streptavidin conjugated to 10 nm colloidal gold. Cells were stimulated with 11 mM glucose plus 100 nM agonist for 1 h, fixed with 2% PFA + 2% glutaraldehyde in 0.1 M cacodylate buffer for 30 min at room temperature, washed with phosphate buffer and prepared for conventional EM. Cells were post-fixed in 3% potassium ferricyanide + 2% osmium tetroxide and treated with tannic acid followed by dehydration with increasing concentrations of ethanol and propylene oxide, embedded in Epon resin and mounted onto Epon stubs. Epon was polymerised overnight at 60 °C and coverslips removed by surface heating. Ultrathin 70 nm sections were cut with a diamond knife on a Leica ultramicrotome, stained with 2% uranyl acetate, visualised in a Tecnai T12 Spirit transmission electron microscope with a CCD camera and analysed in ImageJ (v1.52).

### 4.6. Fluorescent GLP-1R Ligand Uptake Assay in Primary Dispersed Pancreatic Islets

Pancreatic islets were isolated from GLP-1R-iCre-GCaMP3 mice (9–13 weeks old), a mouse strain in which GCaMP3 is restricted to cells with GLP-1R promoter activity [22]. Pancreata were inflated with RPMI-1640 medium containing 1 mg/mL collagenase from *Clostridium histolyticum* (S1745602, Nordmark Biochemicals, Germany), dissected and incubated in a water-bath at 37 °C for 12 min. Islets were subsequently washed and purified using a Histopaque gradient (Histopaque-1119 and -1083). Isolated islets were allowed to recover overnight in RPMI-1640 supplemented with 10% and 1% penicillin/streptomycin. Islet dispersal was achieved by trituration in 0.05% trypsin-EDTA at 37 °C for 3 min, followed by trypsin inactivation with complete medium. Dispersed islet cells were then seeded onto poly-D-lysine-coated coverslips and allowed to adhere overnight in complete RPMI-1640. The following day, 100 nM GLP-1R-TMR, exendin-4-TMR, or vehicle were added to the well, and incubated for 30 min at 37 °C. Medium was then removed, and cells were washed once with HBSS and fixed with 4% PFA for 15 min. Washed coverslips were subsequently mounted onto glass slides in Prolong Diamond anti-fade with DAPI (Thermo Fisher).

### 4.7. High Content Imaging Internalisation and Recycling Assay

INS-1-SNAP-GLP-1R cells in complete medium, or T-REx-SNAP-GLP-1R-SmBiT cells in complete medium ± 0.1 µg/mL tetracycline, were seeded in poly-D-lysine-coated black, clear-bottom plates. Medium was removed and cells labelled with 0.5 µM BG-S-S-649 in complete medium for 20–30 min at 37 °C. After washing three times in HBSS, cells underwent fluorescence imaging to establish the baseline labelling. A 0.75 numerical aperture 20× phase contrast objective was used to acquire epifluorescence and transmitted phase contrast images, with 9 fields-of-view (FOVs) per well. Agonists were then applied in serum-free medium at the indicated dose for a 30 min stimulation period to induce GLP-1R internalisation. Cells were then washed with HBSS, followed by a 5 min treatment ± 100 mM sodium 2-mercaptoethanesulfonate (Mesna) in alkaline TNE buffer (pH 8.6) to cleave residual surface BG-S-S-649 without affecting that internalised whilst bound to SNAP-GLP-1R, re-washed and re-imaged. After a further 60 min recycling period in serum-free medium, a second Mesna treatment was applied to remove any labelled receptor recycled to the cell surface, then cells were washed and re-imaged. Flat-field correction of epifluorescence images was performed using BaSiC [65] and cell segmentation was performed using PHANTAST for the phase contrast image [66]. For quantification of “mean cellular fluorescence intensity”, mean background per image was determined from the segmented epifluorescence image and subtracted from the mean fluorescence intensity from the cell-containing regions. Normalised intensities at internalisation and recycling time-points were expressed as a percentage of the mean intensity at baseline after subtraction of the signal from vehicle-treated cells exposed to Mesna, to establish the degree of specific agonist-induced endocytosis. This was quantified by 3-parameter fitting in Prism 8.0 (Graphpad Software, San Diego, CA, USA) where relevant. Recycling was calculated as the percentage difference between internalisation and recycling time-points.

### 4.8. SNAP-GLP-1R Degradation Assay by Microscopy

INS-1-SNAP-GLP-1R cells were seeded in complete medium in poly-D-lysine-coated black, clear-bottom plates. Medium was removed, cells were washed twice and fresh serum-free medium containing cycloheximide (50 µg/mL) to arrest protein translation was applied. 2 h later, agonists were added in reverse time order, with the medium replaced for the final 30 min of the experiment with complete medium containing 1 µM BG-OG to label total residual SNAP-GLP-1R. Wells were then washed 3 times with HBSS and the microplate was imaged as described in Section 4.7 using a green fluorescence filter set. For T-REx-SNAP-GLP-1R cells, the assay was performed similarly, except cells were seeded in complete medium ± 0.1 µg/mL tetracycline for 18 h to induce GLP-1R expression, which was then removed 2 h prior to the assay to prevent ongoing GLP-1R synthesis. For pulse-chase experiments using INS-1-SNAP-GLP-1R cells, cycloheximide (50 µg/mL) was applied two hours before agonist addition and throughout thereafter. Agonists were applied for 30 min and removed with washing, followed by a 4 h washout period and a 30 min labelling period in complete medium containing 1 µM BG-OG, prior to washing and imaging as in Section 4.7. Where relevant, ECE-1 inhibitor SM-19712 (10 µM; Sigma, St Louis, MO, USA) was supplemented for the duration of the washout period. Total GLP-1R was quantified as the mean cellular fluorescence labelling per well (as defined in Section 4.7), with further subtraction of non-specific fluorescence (i.e., INS-1-SNAP-GLP-1R cells without BG-OG labelling, or T-REx-SNAP-GLP-1R cells labelled with BG-OG but without tetracycline-induced GLP-1R expression). Results were expressed relative to labelled cells without agonist treatment. Time-courses were quantified by calculating the degradation t_1/2_ from one-phase decay exponential curve fitting using Prism 8.0. Pulse-chase experiments were further quantified by 3-parameter fitting of concentration-response data using Prism 8.0.

### 4.9. SNAP-GLP-1R Degradation Assay by Immunoblotting

INS-1-SNAP-GLP-1R cells (1.5 million per dish) were cultured overnight prior to incubation in serum-free medium containing cycloheximide (50 µg/mL) supplemented or not with 10 nM agonist for 6 h. Cells were then washed once in cold PBS and lysed with 1× TNE lysis buffer (20 mM Tris, 150 mM NaCl, 1 mM EDTA, 1% NP40, protease and phosphatase inhibitors) for 10 min at 4 °C. The lysates were then frozen at −80 °C for 2 min, thawed and centrifuged for 10 min at 4 °C at 13,000 rpm. Supernatants were collected, fractionated by SDS-PAGE in urea loading buffer and analysed by western blotting. SNAP-GLP-1R was detected with an anti-SNAP tag rabbit polyclonal antibody (P9310S, New England Biolabs, 1/500) followed by goat anti-rabbit IgG H&L HRP (1/2000, ab6271, Abcam, Cambridge, UK). Post-stripping, tubulin was labelled with anti-α-tubulin mouse monoclonal antibody (T5168, Sigma, 1/5000) followed by sheep anti-mouse secondary antibody HRP (ab6721, Abcam, 1/5000). Blots were developed with the Clarity™ Western ECL Substrate (BioRad, Hercules, CA, USA) in a Xograph Compact X5 processor and specific band densities quantified in Fiji.

### 4.10. Measurement of Binding Affinities

Competition equilibrium binding assays were performed in whole HEK293-SNAP-GLP-1R cells. Cells were labelled with Lumi4-Tb (Cisbio, 40 nM) for 30 min at 37 °C in complete medium. After washing 3 times in HBSS, labelled cells were resuspended in HBSS containing 0.1% BSA, along with metabolic inhibitor cocktail (20 mM 2-deoxyglucose plus 10 mM NaN_3_) to inhibit ATP-dependent endocytosis, as previously described [67], and dispensed into white 96-well plates. After a 30 min preincubation at room temperature, cells were moved to 4 °C before agonist addition. Each experiment included a range of concentrations of the fluorescent antagonist probe exendin (9-39)-FITC [12], along with a range of concentrations of unlabelled test agonists in competition with 10 nM exendin (9-39)-FITC. After a 24 h incubation at 4 °C to reach equilibrium binding, the plate was read by TR-FRET in a Flexstation 3 instrument using the following settings: λ_ex_ = 340 nm, λ_em_ = 490 nm (cut-off 475 nm) and 520 nm (cut-off 515 nm), delay 50 µs, integration 300 µs. Saturation K_d_ for exendin (9-39)-FITC from ratiometric TR-FRET data was established using the “one site–specific binding” algorithm in Prism 8.0 and was used on a per assay basis to determine K_i_ for each test agonist though the “one site–fit K_i_” algorithm. Kinetic binding assays were performed in wild-type and T149M variant T-REx-SNAP-GLP-1R cells after overnight induction of receptor expression using tetracycline (0.1 µg/mL). Cells were labelled and resuspended in HBSS containing BSA and metabolic inhibitors as above. After a 5 min baseline read, a range of concentrations of exendin-4-Cy5 were added and binding was monitored in real time at 37 °C by TR-FRET in a Flexstation 3 instrument using the following settings: λ_ex_ = 340 nm, λ_em_ = 490 nm (cut-off 475 nm) and 668 nm (cut-off 630 nm), delay 50 µs, integration 300 µs. Baseline TR-FRET ratio was subtracted from each well and curve fitting was performed to calculate binding kinetic parameters using the “2 or more concentrations of hot” procedure in Prism 8.0.

### 4.11. NanoBiT Assays

To measure β-arrestin-2 recruitment to GLP-1R, HEK293T cells in 12-well plates were co-transfected for 24 h using Lipofectamine 2000 and the following quantities of DNA: 0.05 µg each of GLP-1R-SmBit and LgBit-β-arrestin-2 with 0.9 µg empty vector DNA (pcDNA3.1). For experiments using stably transfected T-REx-SNAP-GLP-1R cells, 0.2 µg LgBit-β-arrestin-2 with 1.8 µg pcDNA3.1 were transfected per well of a 6-well plate for 36 h, with wild-type or T149M variant receptor expression induced for the final 24 h using tetracycline (0.1 µg/mL). Cells were resuspended in Nano-Glo dilution buffer + furimazine (Promega) diluted 1:20 and seeded in 96-well half area white plates. Baseline luminescence was measured over 5 min using a Flexstation 3 plate reader at 37 °C before addition of ligand or vehicle. After agonist addition, luminescent signal was serially recorded over 20–30 min and normalised to well baseline and to average vehicle-induced signal to establish the agonist effect.

### 4.12. Cyclic AMP Assay

Wild-type or T149M variant GLP-1R expression was induced in T-REx-SNAP-GLP-1R cells by addition of tetracycline (0.1 µg/mL) for 24 h prior to the assay. Cells were treated with a range of concentrations of exendin-4 for 30 min at 37 °C in serum-free DMEM. cAMP was determined by HTRF (Cisbio cAMP Dynamic 2 assay). Curve fitting and bias analysis was performed using Prism 8.0.

### 4.13. Measurement of ECE-1 Activity

ECE-1 activity from INS-1-SNAP-GLP-1R cell lysates was measured using a fluorometric assay (ECE-1 Activity Assay, Promocell, Germany). Lysates prepared from INS-1-SNAP-GLP-1R cells grown in 6-well plates using the provided lysis buffer were diluted 1:5 and ECE-1 activity was determined in duplicate according to the manufacturer’s instructions in the presence or absence of 10 µM SM-19712. Measured activity was expressed relative to sample protein content determined by the BCA assay.

### 4.14. Measurement of Peptide Stability by HPLC

10 nmol GLP-1 or Ex-4 were dissolved in 750 µl ECE buffer (0.2 M Acetate acid buffer, pH 5.5). 0.25 µg recombinant ECE-1 (R&D Systems), or no enzyme as a control for non-enzymatic degradation over the same time period, was added to the reconstituted peptide. Reactions were incubated at 37 °C and 120 µL samples were collected from the reaction vessel at the indicated time-points. 5 µL 10% trifluoroacetic acid (TFA) was added to each sample to terminate enzyme activity. Samples were analysed by reverse-phase high-performance liquid chromatography (HPLC) with a linear acetonitrile/water gradient acidified with 0.1% TFA on Phenomenex Aeris Peptide 3.6 μm XB-C18 Column (150 × 4.6 mm). The eluted peptides were detected at 214 nm. Degradation of peptide was calculated by comparing the area under the peak of the original compound with and without enzyme.

### 4.15. Structural Analysis

The experimental structures of GLP1R were extracted from ProteinDataBank (PDB). The following structures were considered for analysis: 6B3J, 5VAI, 5E94, 5NX2, 3C5T, 3C59, 3I0L, 4ZGM. Using the first two of this list [38,68], mutant GLP-1R structures (GLP-1R-T149M) were generated using an in-house algorithm, as described in Reference [69], and compared to that obtained using another modelling tool, FoldX [70]. Additionally, the experimental structure of β-arrestin-1 bound to the β1-adrenergic receptor [40] was retrieved (PDB ID: 6TK0). The experimental structure of GLP-1R (PDB ID: 6B3J) was superposed to that of the β1-adrenergic receptor and the position of β-arrestin-1 inherited. Manual analysis was performed to assess the following structural features in the wild-type and mutant GLP-1R structure: disulphide bond breakage, steric clash, hydrogen bond breakage, salt bridge breakage, cavity alteration, disallowed phi/psi angles and secondary structure change, as detailed in Reference [69]. For transmembrane (TM) residues, the following structural features were considered: hydrophilic to hydrophobic switch, to proline substitution, charge introduction, charge switch and glycine replacement. Residues and physico-chemical bonds contributing to GLP-1R binding to its ligands were identified using the approach described in Reference [71]. The stability of the wild-type and mutant GLP-1R protein was assessed by calculating the protein-free energy (ΔG, in kcal/mol) using the FoldX algorithm. The amino acid substitution was considered to significantly affect the stability of GLP-1R if the difference in free energy (ΔΔG) between the mutant and wild-type structure was >1 kcal/mol, with ΔΔG > 0 kcal/mol considered destabilising and ΔΔG < 0 kcal/mol stabilizing. An in-house algorithm was used to place the GLP-1R protein structure within the lipid bilayer.

### 4.16. Data Analysis and Statistics

All quantitative data were analysed using Prism 8.0. The indicated *n* refers to the number of independent biological replicates. Technical replicates within the same assay were averaged to determine the mean value for each biological replicate. For experiments with a matched design, paired two-tailed *t*-tests or randomised block analysis of variances (ANOVAs) were performed. Specific statistical tests are indicated in the figure legends. Statistical significance was inferred when *p* < 0.05 without ascribing initial levels of significance at lower *p*-values. Principal component analysis was performed using ClustVis [72] with unit variance scaling applied. Data are represented throughout as mean ± standard error othe mean (SEM), with results from individual biological replicates presented where possible.

## Figures and Tables

**Table 1:** Summary of agonist characteristics. Data are represented as mean ± SEM from *n* = 6 (binding affinity and degradation), *n* = 5 (internalisation and recycling) or *n* = 4 (β-arrestin-2 recruitment) independent experiments.

Assay	N-Terminus	GLP-1	Chi1	Chi2	Chi3	Ex4(1-30)	Ex4	GLP-1-G2
Log K_i_ (M)	H1	−7.6 ± 0.1	−8.2 ± 0.1	−8.4 ± 0.1	−9.1 ± 0.0	−9.0 ± 0.0	−9.5 ± 0.1	−7.0 ± 0.1
	F1	−6.4 ± 0.1	−6.4 ± 0.1	−6.5 ± 0.0	−7.8 ± 0.1	−7.5 ± 0.0	−7.3 ± 0.1	−6.1 ± 0.0
	D3	−8.0 ± 0.1	−8.5 ± 0.1	−9.0 ± 0.1	8.6 ± 0.1	−9.2 ± 0.1	−9.2 ± 0.1	−7.6 ± 0.1
β-arrestin-2 recruitment (AUC)	H1	6.2 ± 0.9	7.0 ± 1.6	8.4 ± 2.3	8.9 ± 2.1	4.0 ± 0.5	10.2 ± 1.6	1.3 ± 0.5
	F1	2.0 ± 1.3	2.0 ± 0.8	4.6 ± 1.6	4.8 ± 1.4	0.8 ± 0.2	1.3 ± 1	0.9 ± 0.5
	D3	4.9 ± 0.9	9.4 ± 0.5	8.1 ± 1.2	8.9 ± 1.4	8.8 ± 0.3	8.3 ± 0.3	4.0 ± 0.7
Internalisation (%)	H1	69.6 ± 14.1	88.9 ± 12.7	97.3 ± 14.3	93 ± 13.7	84.5 ± 17.4	110.7 ± 9.9	58.9 ± 11.4
	F1	28.1 ± 11.1	18.5 ± 8.7	24.4 ± 9.3	40.6 ± 19.4	29.1 ± 11.3	25.5 ± 11.8	12.7 ± 7.8
	D3	88.5 ± 16.9	97.7 ± 14.1	78.6 ± 15.7	101.5 ± 12.2	98 ± 12.3	105.4 ± 11.7	60.3 ± 17
Recycling (% internalised GLP-1R)	H1	44.1 ± 3.1	35.3 ± 2.8	30.9 ± 3.2	21.8 ± 3.8	26.4 ± 2.8	20.8 ± 3.6	44.4 ± 3.8
	F1	55.2 ± 3.4	48.7 ± 4.8	48.5 ± 5.2	40.7 ± 2.7	45.9 ± 3.6	49.9 ± 3.2	62.7 ± 17.9
	D3	33.2 ± 4.1	28.6 ± 4.0	22.9 ± 5.1	19.0 ± 4.3	19.2 ± 3.2	19.0 ± 3.5	35.4 ± 5.6
Degradation (% remaining GLP-1R)	H1	80.4 ± 2.7	72.9 ± 1.6	69.1 ± 5.7	52.8 ± 5.9	57.0 ± 5.3	52.6 ± 6.6	86.2 ± 5.5
	F1	96.4 ± 6.7	95.6 ± 2	92.8 ± 4.5	77.1 ± 6.6	91.8 ± 6.4	99.3 ± 5.0	102.8 ± 4.7
	D3	58.9 ± 4.4	62.9 ± 3.8	51.3 ± 8.1	51.8 ± 7.7	51.9 ± 7.1	55.0 ± 4.2	62.2 ± 7.7

## Supplementary Materials

The following are available online at https://www.mdpi.com/1422-0067/21/21/8404/s1.
