# Supplementary material for: Ligand-Specific Factors Influencing GLP-1 Receptor Post-Endocytic Trafficking and Degradation in Pancreatic Beta Cells
Source: Int J Mol Sci. 2020 Nov 9;21(21):8404. doi: 10.3390/ijms21218404 (PMC7664906; doi:10.3390/ijms21218404)

Supplementary Materials

Ligand-specific factors influencing GLP-1 receptor post-endocytic trafficking and degradation in pancreatic beta cells

Zijian Fang[1,2], Shiqian Chen[1], Yusman Manchanda[3], Stavroula Bitsi[3], Philip Pickford[1], Alessia David[4], Maria M Shchepinova[5], Ivan R Corrêa Jr[6], David J Hodson[7,8], Johannes Broichhagen[9,10], Edward W Tate[5], Frank Reimann[11], Victoria Salem[1], Guy A Rutter[3,12], Tricia Tan[1], Steven R Bloom[1], Alejandra Tomas*[,3], Ben Jones*[,1].

[1] Section of Endocrinology and Investigative Medicine, Imperial College London, London W12 0NN, United Kingdom.

[2] Current address: Wellcome Trust – Medical Research Council Cambridge Stem Cell Institute and Department of Haematology, University of Cambridge, Cambridge CB2 0AW, United Kingdom.

[3] Section of Cell Biology and Functional Genomics, Imperial College London, London W12 0NN, United Kingdom.

[4] Centre for Bioinformatics and System Biology, Department of Life Sciences, Imperial College London.

[5] Department of Chemistry, Imperial College London, Molecular Sciences Research Hub, 80 Wood Lane, London, W12 0BZ, United Kingdom.

[6] New England Biolabs, Ipswich, USA.

[7] Institute of Metabolism and Systems Research (IMSR), and Centre of Membrane Proteins and Receptors (COMPARE), University of Birmingham, Birmingham, United Kingdom.

[8] Centre for Endocrinology, Diabetes and Metabolism, Birmingham Health Partners, Birmingham, United Kingdom.

[9] Department of Chemical Biology, Forschungsinstitut für Molekulare Pharmakologie, Berlin, Germany.

[10] Department of Chemical Biology, Max Planck Institute for Medical Research, Heidelberg, Germany.

[11] Institute of Metabolic Science & MRC Metabolic Diseases Unit, University of Cambridge, Cambridge, Addenbrooke's Hospital, Hills Road, Cambridge CB2 0QQ, United Kingdom.

[12] Lee Kong Chian School of Medicine, Nanyang Technological University, Singapore.

* Co-senior and corresponding authors

Contents:

- Supplementary Figure 1: Fluorescent ligands used in this study
- Supplementary Figure 2: BG-OG labelling.
- Supplementary Figure 3: Structural analysis of T149M variant
- Supplementary Methods (General Chemistry)
- Synthesis of Ex4(1-39)_S39C_Cy5

1. Supplementary Figure 1

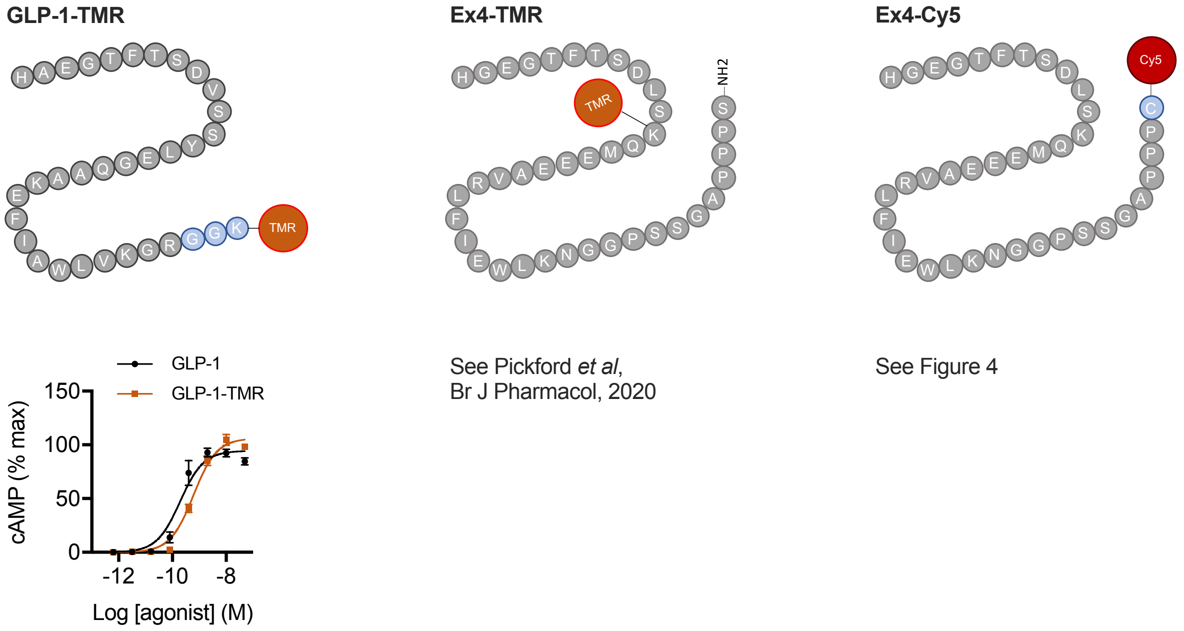

Fluorescent ligands used in this study. Amino acid sequence of GLP-1-TMR, Ex4-TMR and Ex4-Cy5, with fluorophore position indicated. Linker sequences are shown in blue. cAMP production in HEK293-SNAP-GLP-1R cells with unmodified GLP-1 *versus* GLP-1-TMR, 30 min stimulation, *n*=4, with 3-parameter fit shown. The equivalent analysis for Ex4-TMR is found in Pickford *et al*., Br J Pharmacol, 2020. Binding affinity for Ex4-Cy5 is found in Figure 4 of the present work.

2. Supplementary Figure 2

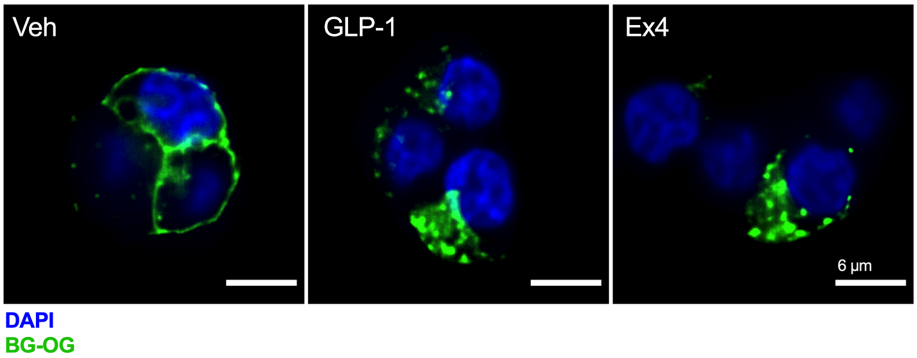

BG-OG labelling. Representative images from *n*=2 experiments of INS-1-SNAP-GLP-1R cells treated with 100 nM GLP-1 or exendin-4 for 30 min with concurrent labelling with BG-OG labelling (0.5 µM).

3. Supplementary Figure 3

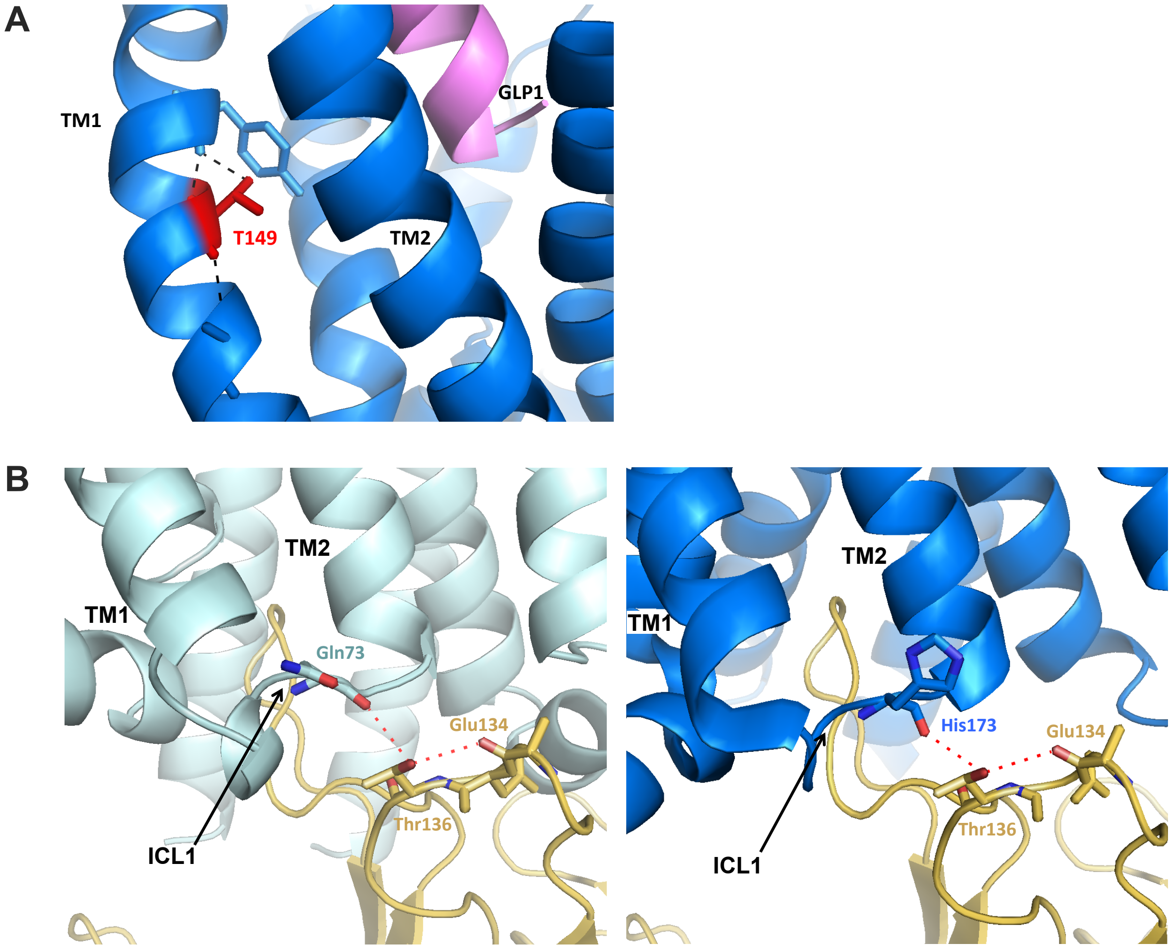

Structural analysis of T149M variant. (**A**) T149 structural role assessed using the experimental structure of GLP-1R in complex with GLP-1. The intrahelical hydrogen bond formed by T149 (in red) is highlighted with black dashes. The GLP-1R is presented in blue and the GLP-1 in magenta. TM, transmembrane (**B**) β-arrestin-1-GPCR interaction. On the left, experimental structure of β-arrestin-1 (presented in yellow) bound to the β1-adrenergic receptor (coloured in cyan); on the right, the predicted structure of β-arrestin-1 bound to GLP-1R (coloured in blue). The position of the transmembrane helices 1 (TM1) and 2 (TM2) and of the first intracellular loop (ICL1) of the beta1 adrenoceptor and the GLP1R are presented. Polar interaction between residues on the ICL1 and the middle loop of arrestin are shown with dotted red lines.

4. Supplementary Methods

General chemistry

RP-HPLC was performed on a Waters e2695 system equipped with a 2998 PDA detector for product collection (at 650 nm) on a Supelco Ascentis® C18 HPLC Column (5 µm, 250 × 10 mm). Buffer A: 0.1% TFA in H_2O Buffer B: acetonitrile. The typical gradient was from 10% B for 5 min → gradient to 90% B over 45 min → 90% B for 5 min → gradient to 99% B over 5 min with 4 mL/min.

High resolution mass spectrometry was performed using a Bruker maXis II ETD hyphenated with a Shimadzu Nexera system. The instruments were controlled *via* Brukers otofControl 4.1 and Hystar 4.1 SR2 (4.1.31.1) software. The acquisition rate was set to 3 Hz and the following source parameters were used for positive mode electrospray ionization: End plate offset = 500 V; capillary voltage = 3800 V; nebulizer gas pressure = 45 psi; dry gas flow = 10 L/min; dry temperature = 250 °C. Transfer, quadrupole and collision cell settings are mass range dependent and were fine-adjusted with consideration of the respective analyte's molecular weight. For internal calibration sodium format clusters were used. Samples were desalted *via* fast liquid chromatography. A Supelco Titan™ C18 UHPLC Column, 1.9 µm, 80 Å pore size, 20 × 2.1 mm and a 2 min gradient from 10 to 98% aqueous MeCN with 0.1% FA (H_2O: Carl Roth GmbH + Co. KG ROTISOLV® Ultra LC-MS; MeCN: Merck KGaA LiChrosolv® Acetonitrile hypergrade for LC-MS; FA - Merck KGaA LiChropur® Formic acid 98%-100% for LC-MS) was used for separation. Sample dilution in 10% aqueous MeCN (hyper grade) and injection volumes were chosen dependent of the analyte's ionization efficiency. Hence, on-column loadings resulted between 0.25–5.0 ng. Automated internal re-calibration and data analysis of the recorded spectra were performed with Bruker's DataAnalysis 4.4 SR1 software.

5. Synthesis of Ex4(1-39)_S39C_Cy5

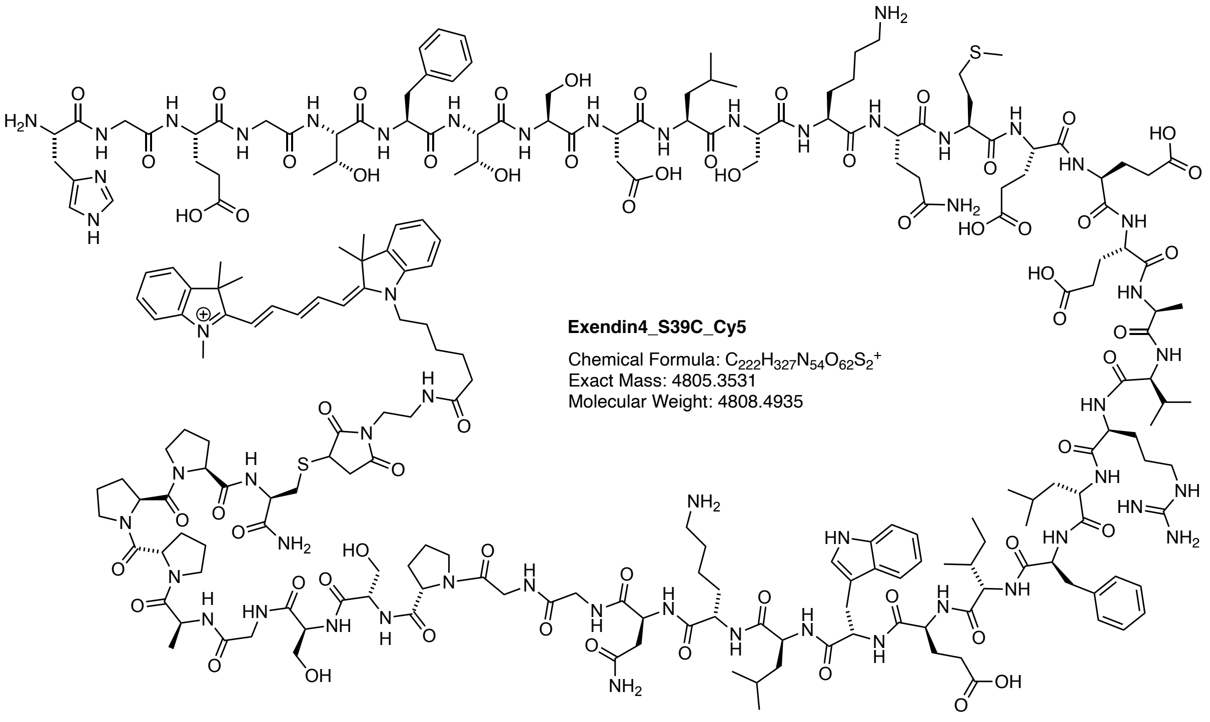

To a solution of Ex4(1-39)_S39C (Wuxi Apptec, 900 μg, 214 nmol, 1.0 eq.) in PBS (500 μL) was added Cy5-Mal (Lumiprobe #43020, 321 nmol, 1.5 eq.). The solution was incubated on a shaker (500 rpm) at 37 °C over night before being subjected to RP-HPLC purification. The purified fractions were combined, the concentration was determined *via* the absorption of the Cy5 fluorophore at 647 nm (ε = 250,000 M^{-1} cm^{-1} in PBS) and aliquots of 5 nmol each were prepared and lyophilized to yield **Ex4(1-39)_S39C_Cy5** as a deep blue powder.

HRMS (ESI): calc. for $C_{222}H_{332}N_{54}O_{62}S_2$ $[M+5H]^{6+}$: 802.0658, found: 802.0659.

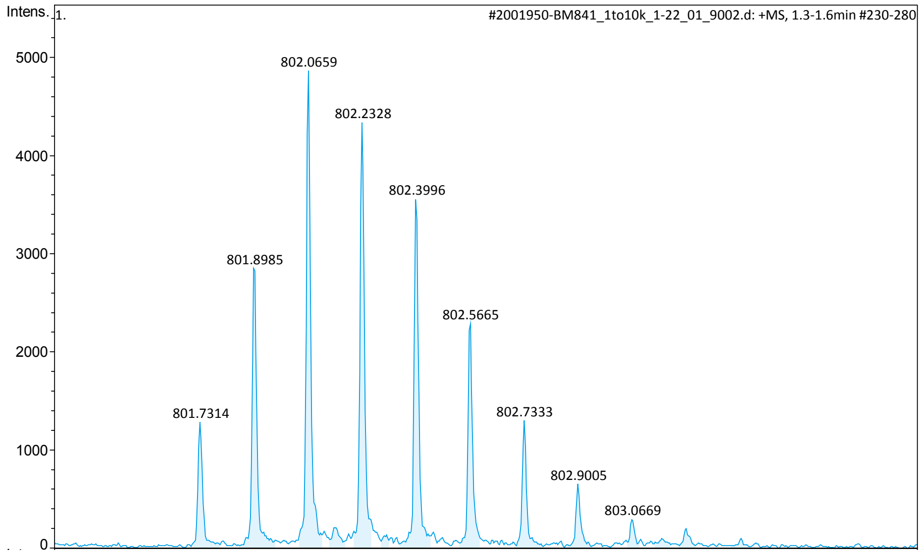

Supplement: Supplementary file 1 [file ijms-21-08404-s001.pdf]
